# Mechanical Induction of PGE_2_ in Osteocytes Blocks Glucocorticoid-Induced Apoptosis Through Both the β-Catenin and PKA Pathways

**DOI:** 10.1002/jbmr.168

**Published:** 2010-07-24

**Authors:** Yukiko Kitase, Leonardo Barragan, Hai Qing, Shino Kondoh, Jean X Jiang, Mark L Johnson, Lynda F Bonewald

**Affiliations:** 1 Department of Oral Biology, School of Dentistry, University of Missouri at Kansas City Kansas City, MO, USA; 2 Department of Oral Biological and Medical Sciences, Faculty of Dentistry, University of British Columbia Vancouver, British Columbia, Canada; 3 Institute of Molecular and Cellular Biosciences, University of Tokyo Tokyo, Japan; 4 Department of Biochemistry, University of Texas Health Science Center San Antonio, TX, USA

**Keywords:** OSTEOCYTES, MLO-Y4 CELLS, APOPTOSIS, PGE_2_, WNT SIGNALING

## Abstract

Glucocorticoids are known to induce osteocyte apoptosis, whereas mechanical loading has been shown to sustain osteocyte viability. Here we show that mechanical loading in the form of fluid-flow shear stress blocks dexamethasone-induced apoptosis of osteocyte-like cells (MLO-Y4). Prostaglandin E_2_ (PGE_2_), a rapidly induced signaling molecule produced by osteocytes, was shown to be protective against dexamethasone-induced apoptosis, whereas indomethacin reversed the antiapoptotic effects of shear stress. This protective effect of shear stress was mediated through EP2 and EP4 receptors, leading to activation of the cAMP/protein kinase A signaling pathway. Activation of phosphatidylinositol 3-kinase, an inhibitor of glycogen synthesis kinase 3, also occurred, leading to the nuclear translocation of β-catenin, an important signal transducer of the Wnt signaling pathway. Both shear stress and prostaglandin increased the phosphorylation of glycogen synthesis kinase 3 α/β. Lithium chloride, an activator of the Wnt pathway, also was protective against glucocorticoid-induced apoptosis. Whereas it is known that mechanical loading increases cyclooxygenase-2 and EP2 receptor expression and prostaglandin production, dexamethasone was shown to inhibit expression of these components of the prostaglandin pathway and to reduce β-catenin protein expression. β-catenin siRNA knockdown experiments abrogated the protective effects of PGE_2_, confirming the central role of β-catenin in mediating the protection against dexamethasone-induced cell death. Our data support a central role for PGE_2_ acting through the cAMP/PKA and β-catenin signaling pathways in the protection of osteocyte apoptosis by fluid-flow shear stress. © 2010 American Society for Bone and Mineral Research.

## Introduction

Glucocorticoid-induced osteoporosis is the second most common form of osteoporosis in the United States.(1) Individuals treated with glucocorticoids have alterations in bone remodeling, including an increase in bone resorption and suppression of bone formation.(2) Reductions in trabecular bone mass and trabecular thickness and number are thought to be responsible for increased bone fragility.(3–5) These reductions in bone mass are thought to be due to an increase in both osteoblast and osteocyte cell death, or apoptosis. It also has been proposed that the increased fragility in these patients compared with postmenopausal osteoporotic patients is due to changes in the localized material properties of trabecular bone surrounding osteocyte lacunae.(6)

The most abundant bone cell type is the osteocyte (90% to 95% of all bone cells), terminally differentiated osteoblasts embedded in the mineralized matrix.(7) Osteocytes are proposed to coordinate osteoblast and osteoclast activity in response to mechanical stimuli, translating mechanical strain into biochemical signals that ultimately regulate resorption or formation.(8,9) Osteocyte cell death is thought to signal osteoclast recruitment and activation, leading to bone loss,(10,11) and recently, this has been validated in vivo through the use of targeted deletion of osteocytes.(12) Osteocyte cell death can occur in association with age and pathologic conditions such as osteoporosis and osteoarthritis, leading to increased bone fragility.(13,14) Such fragility may be due to loss of the ability of the osteocyte to sense microdamage and signal to other bone cells for repair.(15) Hence osteocyte viability appears to play a significant role in the maintenance of bone homeostasis and integrity.

Mechanical loading can maintain or increase bone mass, whereas unloading or immobilization results in loss of bone mass. Mechanical loading has been reported to play a role in maintaining osteocyte viability. Reduced mechanical loading in vivo increases the number of osteocytes undergoing apoptosis.(16) Shear stress inhibits osteocyte apoptosis induced by low serum,(17) and dexamethasone-induced osteocyte apoptosis is attenuated by a form of mechanical loading known as *substrate stretching*.(18) Fluid-flow shear stress (FFSS) is most likely the form of loading to which the osteocyte is exposed.(19–21) Primary osteocytes have been shown to be more sensitive to shear stress than osteoblasts or fibroblasts and to substrate stretching compared with hydrostatic compression.(22) The biochemical signals induced by mechanical loading responsible for maintaining osteocyte viability are not known. However, it has been shown known that fluid-flow shear stress applied to MLO-Y4 osteocytes results in the release of PGE_2_, and this leads to activation of β-catenin signaling.(23–25) The importance of β-catenin signaling in the prevention of apoptosis is well established in several other systems,(26–28) and this raises the possibility that activating this pathway in osteocytes could have a similar protective effect.

This study was undertaken to determine if mechanical loading in the form of fluid-flow shear stress could inhibit glucocorticoid-induced osteocyte apoptosis and, if so, to identify the factor(s) responsible and determine the molecular signaling mechanisms responsible for this protection. Here we show that shear stress prevents osteocyte apoptosis induced by dexamethasone. The protective effect was mediated by prostaglandin E_2_ (PGE_2_) through both the classic cAMP/protein kinase A (PKA) pathway and crosstalk between the phosphatidylinositol 3-kinase (PI3K)/Akt and β-catenin signaling pathways in osteocyte-like MLO-Y4 cells. Dexamethasone inhibited β-catenin stabilization and the expression of its target genes in addition to its inhibitory effects on cyclooxygenase-2 (COX-2) and EP2 receptor gene expression and PGE_2_ production. This study may have important implications for the treatment of glucocorticoid-induced osteoporosis because the pathways used by shear stress contain molecular elements that could serve as therapeutic targets.

## Materials and Methods

### Cell culture

The osteocyte-like MLO-Y4 cell line, derived from murine long bone,(29) was used as an in vitro osteocyte model. MLO-Y4 cells were cultured on collagen type I–coated plates or glass slides (rat tail collagen type I, 0.15 mg/mL) in α-MEM without phenol red supplemented with 2.5 % fetal bovine serum (FBS), 2.5% calf serum (CS) in a 5% CO_2_ incubator at 37°C.

### Fluid-flow shear-stress (FFSS) experiment

MLO-Y4 cells were plated on glass slides coated with type I collagen at a density of 3 × 10^4^ cells/cm^2^ and were used at approximately 80% confluence. Cells were subjected to 16 dyn/cm^2^ steady laminar FFSS for 2 hours in the presence or absence of 1 µM indomethacin. After incubation for 2 hours, 1 µM dexamethasone (Sigma-Aldrich, St Louis, MO, USA) was added for an additional 6 hours to induce apoptosis. Pretreatment was performed with indomethacin 12 hours before application of FFSS, during FFSS, and during the 2-hour postincubation time. Cells were fixed with neutral buffered formalin and stained with DAPI. Cells that showed nuclear condensation, blebbing, and fragmentation were counted as apoptotic cells. Four fields per each slide and 500 cells were counted from each field under the microscope (*n* = 3).

### Quantification of apoptotic cells

Apoptotic cells were quantified by nuclear fragmentation assay and trypan blue exclusion assay, as described previously.(30) MLO-Y4 cells were plated at 1 × 10^4^ cells/cm^2^ on a collagen-coated 48-well plate, with three to four wells used for each experimental condition. Representative examples of each assay are shown. Cells were pretreated with varying concentrations of PGE_2_ (Sigma-Aldrich), 5 µM butaprost, 5 µM sulprostone, 5 µM PGE_1_ alcohol (Cayman Chemical, Ann Arbor, MI, USA), 100 µM 8-bromo-cAMP (Sigma-Aldrich), or 10 mM LiCl for 1 hour, followed by treatment with 1 µM of dexamethasone for 6 hours. If necessary, cells were pretreated with 5 µM of EP2 antagonist AH6809 (6-isopropoxy-9-oxoxanthene-2-carboxylic acid; Cayman Chemical), 5 µM of EP4 antagonist CP-147499 (kindly provided by Dr Lydia Pan, Pfizer, Inc., Groton, CT, USA), 5 µM of H89 (isobutylmethylxanthine; Sigma-Aldrich), or 1 µM of wortmannin (Sigma-Aldrich) for 0.5 to 1 hour prior to addition of PGE_2_. For the nuclear fragmentation assay, MLO-Y4 cells were stained with DAPI. Cells exhibiting chromatin condensation and nuclear fragmentation were detected by fluorescence microscopy. A total of 500 cells were examined for each experimental condition by systematic random sampling. The percentage of MLO-Y4 cells stained with trypan blue has been shown previously to correlate with that of apoptotic cells.(30) For the trypan blue assay, after treatment, adherent cells released by trypsin-EDTA were combined with nonadherent cells and collected by centrifugation. Then 0.04% trypan blue (Sigma-Aldrich) was added, and cells exhibiting both nuclear and cytoplasmic staining were determined using a hemocytometer under a light microscope. A total of 100 cells per each experimental condition were counted.

### Western blot analysis

For Western blot studies, MLO-Y4 cells were grown on type I collagen–coated 6-well plates or glass slides at 1 × 10^4^ cells/cm^2^. At the various indicated time points, cells were treated with (1) 16 dyn/cm^2^ FFSS or (2) incubated with 5 µM of PGE_2_ in the presence or absence of preincubation with wortmannin or H89 for 0.5 to 1 hour. As a positive control, cells were treated with 10 mM of LiCl, which inhibits glycogen synthesis kinase 3 (GSK-3). After each treatment, the cells were washed with cold PBS twice and lysed with RIPA buffer including proteinase and phosphatase inhibitors (Sigma-Aldrich). The lysates were sheared using a 22-gauge needle, centrifuged at 12,000 rpm for 10 minutes at 4°C, and the supernatants were collected. The cell lysate and sample buffer were mixed and boiled for 5 minutes before loading on the gel. Proteins (5 µg) were separated by SDS-PAGE under constant voltage (160 V) and were transferred electrophoretically to a nitrocellulose membrane (Bio-Rad, Hercules, CA, USA) at a 60-V constant current for 2 hours. The membranes were blocked in a blocking solution overnight at 4°C and incubated with the primary antibody [anti-phospho-GSK3α/β (1:1000; R&D Systems, Minneapolis, MN, USA), anti-GSK-3α, anti-GSK-3β (1:1000; Cell Signaling Technology, Danvers, MA, USA), anti-β-catenin (1:4000; Abcam, Cambridge, MA, USA), or anti-actin (1:4000; Sigma-Aldrich)] overnight at 4°C. The blots were incubated with a horseradish peroxidase–linked secondary antibody (antirabbit/antimouse IgG; Boehringer, Mannheim, Germany) for 2 hours at a room temperature. Afterwards, the immunoblots were visualized with a chemiluminescence detection kit (Pierce, Rockford, IL, USA). The semiquantitative analysis of band intensity was performed using an EPSON scanner and NIH Image 1.63 software. The intensity of GSK-3α/β total protein and actin was used for normalization of phosphorylated GSK-3α/β.

### Immunofluorescence

MLO-Y4 cells were grown on type I collagen–coated glass slides at 1 × 10^4^ cells/cm^2^ and then treated with 16 dyn/cm^2^ FFSS or 5 µM of PGE_2_ for 2 hours. As a positive control, cells were treated with 10 mM of LiCl. After each treatment, the cells were washed with cold PBS twice, fixed in cold 4% paraformaldehyde–0.02% Triton for 5 minutes, and washed three times with PBS. The slides were blocked with a blocking solution overnight at 4°C and incubated with the primary antibody against β-catenin (E-17) (1:100, Santa Cruz Biotechnology, Inc., Santa Cruz, CA, USA) overnight at 4°C in a humidified chamber. After washing with PBS three times, a Cy3-labeled secondary donkey antigoat IgG antibody (1:200; Jackson, ImmunoResearch, West Grove, PA, USA) was incubated on the sections for 1 hour at room temperature and then washed with PBS six times. Coverslips were mounted with mounting medium (9:1 glycerol/PBS plus 5% *N*-propyl gallate). Digital images were acquired with an optronics camera and analyzed with imager software. As negative controls, the primary antibody was omitted from the staining procedure.

### mRNA isolation and microarray analysis

Gene array analysis was performed on MLO-Y4 cells treated with dexamethasone for 4 and 24 hours. After each treatment, total RNA was isolated from cells using TRIzol Reagent (Invitrogen, Carlsbad, CA, USA). cDNA was made and denatured, labeled, and then hybridized to the mouse genome 430A 2.0 array chips (Affymetrix, Santa Clara, CA, USA). A set of microarray hybridization experiments was performed according to the manufacturer's protocols. After hybridization, microarray experiments were analyzed for expression changes based on the GCOS package with no statistical evaluation except the default with one array per condition and present and absent calls of the software.

### Quantitative real-time polymerase chain reaction (qRT-PCR)

In addition, 1 × 10^4^ cells/cm^2^ of MLO-Y4 cells were incubated in 100-mm tissue culture plates in the presence or absence of 1 µM of dexamethasone for 6, 24, and 48 hours. After treatment, total RNA was extracted using TRIzol Reagent (Invitrogen) and reverse transcribed at 48°C for 30 minutes using a Taqman Reverse Transcription Reagents Kit (Applied Biosystems, Austin, TX, USA) in a final volume of 20 µL. Two microliters of cDNA were used as template for amplification by PCR. Taqman real-time quantitative PCR (qPCR) analysis for COX-2 (Applied Biosystems, Cat. No. Mm01307334_g1), EP2 receptor (Applied Biosystems, Cat. No. Mm00436051_m1), and glyceraldehyde-3-phosphate dehydrogenase (GAPDH; Applied Biosystems, Cat. No. 4308313) was performed as described by the manufacturer's protocol using premade Taqman gene expression assays (Applied Biosystems). *GAPDH* expression levels were used as reference for normalization.

### *β-Catenin* siRNA experiments

MLO-Y4 cells were plated at 1 × 10^4^ cells/cm^2^ on collagen-coated 6-well plates 1 day prior to the start of the *β-catenin* siRNA experiment. Cells were transiently transfected with siRNA oligonucleotides (50 nM) using Oligofectamine Reagent (Invitrogen). *β-Catenin* siRNA and negative-control RNA were purchased from Ambion (Austin, TX, USA). Reduction of *β-catenin* mRNA levels was measured by qPCR 24 hours after transfection, and protein levels were analyzed by Western blotting 48 hours after transfection. At 48 hours after transfection, the cells were exposed to 5 µM of PGE_2_ for 2 hours, followed by 1 µM of dexamethasone for 6 hours. After treatment, apoptotic cells were determined by trypan blue staining, as described earlier. A total of 100 cells per each experimental condition were counted.

### Enzyme-linked immunosorbent assay (ELISA)

MLO-Y4 cells were incubated in 48-well plates at 1 × 10^4^ cells/0.3 mL of medium per well in the presence or absence of 1 µM dexamethasone for 24 and 48 hours. After treatment, supernatants were collected, and the concentrations of soluble PGE_2_ were assayed using a commercially available kit (Prostaglandin E_2_ EIA, Cayman Chemical), according to manufacturer's protocols.

### Statistical analysis

All data are presented as mean ± SD and *n* = 3 to 4. The statistical significance of difference between mean values was determined by one-way ANOVA followed by Tukey-Kramer post hoc analysis. All comparisons at *p* < .05 were considered significant.

## Results

### FFSS is protective against dexamethasone-induced osteocyte apoptosis owing to a soluble factor and indomethacin abrogates the protective effect of FFSS

Since mechanical loading has been reported to play an important role in maintaining osteocyte viability, we performed nuclear fragmentation and trypan blue exclusion assays to examine the effect of FFSS on apoptosis in MLO-Y4 osteocyte-like cells (Table 1). Treatment of MLO-Y4 cells with 1 µM of dexamethasone for 6 hours resulted in an approximately 50% increase in the number of apoptotic cells measured by either nuclear fragmentation or trypan blue exclusion. Two hours of 16 dyn/cm^2^ steady FFSS effectively protected against the dexamethasone-induced apoptosis. Since FFSS is well known to rapidly induce release of prostaglandins from osteocytes(31) and MLO-Y4 cells,(32) we examined the effects of indomethacin, a potent inhibitor of prostaglandin synthesis. In the presence of 1 µM of indomethacin, present during the entire experimental time, the protective effects of FFSS were significantly abrogated. In addition, harvested conditioned media from cells subjected to FFSS blocked dexamethasone-induced apoptosis, indicating that a soluble factor produced by MLO-Y4 cells might be mediating the protective effect of FFSS (Table 1).

**Table 1:** Effect of Dexamethasone, FFSS, PGE_2_, and Indomethacin and Fluid Flow Conditioned Media (FFCM) on Apoptosis of MLO-Y4 Osteocytes

Nuclear fragmentation (apotosis %)	
Control	8.75 ± 0.80
Dexamethasone	12.78 ± 0.45^a^
FFSS + dexamethasone	9.03 ± 0.85^b^
Indo/FFSS/dexamethasone	12.99 ± 0.35^a^
Trypan blue exclusion (dead cell %)	
Control	7.24 ± 0.64
Dexamethasone	11.37 ± 1.24^a^
FFCM + dexamethasone	7.13 ± 1.31^b^
PGE_2_ + dexamethasone	9.31 ± 0.60^b^

a *p* < .01 versus control.

b *p* < .01 versus dexamethasone.

### Exogenous addition of PGE_2_ protects against dexamethasone-induced MLO-Y4 cell apoptosis

Since indomethacin effectively abrogated the protective effect of FFSS on dexamethasone-induced apoptosis (Table 1), we next examined whether exogenous addition of PGE_2_ would have the same antiapoptotic protective effect using both the nuclear fragmentation assay and trypan blue exclusion assay and to determine the optimal dose response (Table 2). Both assays revealed that PGE_2_ (10^−5^ to 10^−8^ M) was able to prevent or reduce MLO-Y4 cell apoptosis induced by dexamethasone. Taken together, these data support the hypothesis that PGE_2_ release in response to FFSS is protective against glucocorticoid-induced osteocyte apoptosis.

**Table 2:** PGE_2_ Dose-Dependent Decrease in Dexamethasone-Induced MLO-Y4 Cell Apoptosis

Condition	Nuclear fragmentation (% apoptotic cells)	Trypan blue (% dead cells)
Control	11.45 ± 1.34	8.48 ± 0.63
Dexamethasone	18 ± 1.71^a^	17.22 ± 2.66^a^
Dexamethasone + 10^−8^ PGE_2_	15.4 ± 0.35^b^ ^c^	13.80 ± 1.14^a^ ^c^
Dexamethasone + 10^−7^ PGE_2_	15.13 ± 0.31^b^ ^c^	11.77 ± 0.63^a^ ^d^
Dexamethasone + 10^−6^ PGE_2_	10.67 ± 0.92^d^	9.75 ± 2.53^d^
Dexamethasone + 10^−5^ PGE_2_	nd	9.11 ± 1.01^d^

a *p* < .01 versus control.

b *p* < .05 versus control.

c *p* < .05 versus dexamethasone.

d *p* < .01 versus dexamethasone.

### The protective effect of PGE_2_ is mediated through EP2 and EP4 receptors

**Fig. 1:**
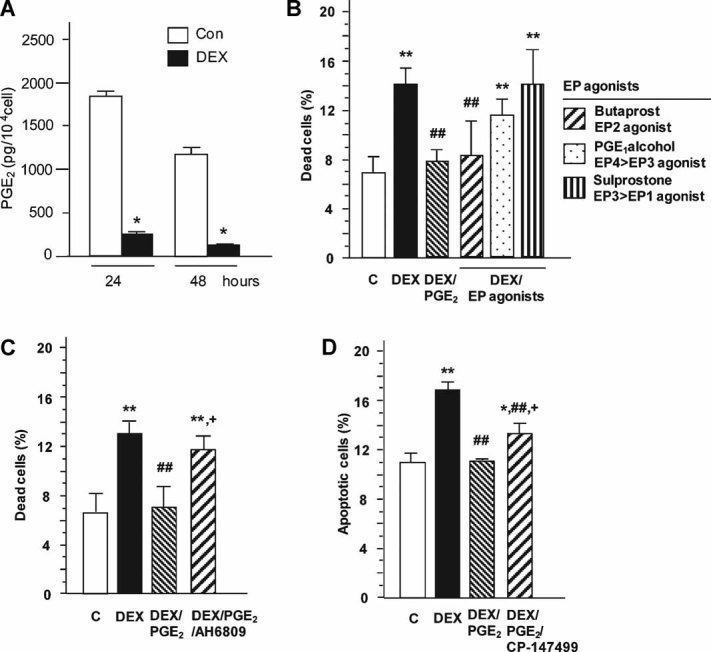
(*A*) The effects of dexamethasone on PGE_2_ production by MLO-Y4 cells. PGE_2_ concentration in MLO-Y4 cells was measured by EIA (Cayman Chemicals). *p < .05. (*B–D*) The effects of PGE_2_ on dexamethasone-induced apoptosis are mediated through the EP2 and EP4 receptors. (*B*) MLO-Y4 cells were pretreated with 5 µM of PGE_2_, 5 µM butaprost (EP2 agonist), 5 µM of PGE_1_ alcohol (EP3/EP4 agonist), or 5 µM of sulprostone (EP1/EP3 agonist) for 1 hour followed by 1 µM of dexamethasone for 6 hours. Treatment with butaprost significantly inhibited dexamethasone-induced cell death to a similar extent as PGE_2_. To further confirm the EP receptor mediating the effect of PGE_2_, MLO-Y4 cells were pretreated with AH6809, an EP2-selective antagonist (*C*), or CP-147499, an EP4 selective antagonist (*D*), for 30 minutes, followed by treatment with 5 µM of PGE_2_ for 1 hour. The cells then were exposed to 1 µM of dexamethasone for 6 hours. AH6809 reversed the protective effect of PGE_2_ (*C*), whereas CP-147499 only reversed the protective effect of PGE_2_ slightly (*D*). Panels *A–C:* **p < .01 and *p < .05 versus control; ^##^*p* < .01 versus dexamethasone alone and ^+^*p* < .05 versus dexamethasone with pretreatment of PGE_2_.

Since PGE_2_ was able to protect against the effects of dexamethasone, it was logical to ask whether dexamethasone affected intracellular stores of PGE_2_ in MLO-Y4 cells and whether (which) PGE_2_ receptors mediated the PGE_2_ protection. As shown in Fig. 1*A*, MLO-Y4 cells treated with dexamethasone for 24 and 48 hours produced 7.5 (control 1854 ± 57, dexamethasone 246 ± 23^a^ pg/10^4^ cells) and 9 (control 1174 ± 67, dexamethasone 131 ± 4^a^ pg/10^4^ cells) fold less PGE_2_, respectively, compared with control, as determined by ELISA (*p* < .001 versus each control). Next, we determined which receptor is responsible for the protection of PGE_2_ against osteocyte apoptosis. PGE_2_ is known to exert its effects through four different types of receptors, EP1, EP2, EP3, and EP4.(33) Therefore, we examined the effect of pharmacologic activators and inhibitors of EP receptors on osteocyte cell death (Fig. 1*B–D*). Addition of 5 µM of butaprost, an EP2 agonist, prevents dexamethasone-induced cell death to a similar extent as 5 µM of PGE_2_. However, 5 µM of sulprostone, an EP1/EP3 agonist, did not inhibit osteocyte cell death. Similarly, 5 µM of PGE_1_ alcohol, another EP3/EP4 receptor agonist, did not statistically inhibit cell death (Fig. 1*B*). These data suggest that the protective effect of PGE_2_ is mediated through EP2 and EP4, but not EP1 and EP3 receptors. To further verify the involvement of EP2 and EP4 receptors in the protective function of PGE_2_, selective antagonists against each receptor were tested. Five micromolar AH6809, an EP2 selective antagonist, partially but significantly reversed the protective effect of PGE_2_ (Fig. 1*B*). Five micromolar CP-147499, an EP4 selective antagonist (Fig. 1*D*), was less effective than the EP2 selective antagonist AH6809 (Fig. 1*C*).

### The cAMP/PKA pathway partially mediates the protective effect of PGE_2_

**Fig. 2:**
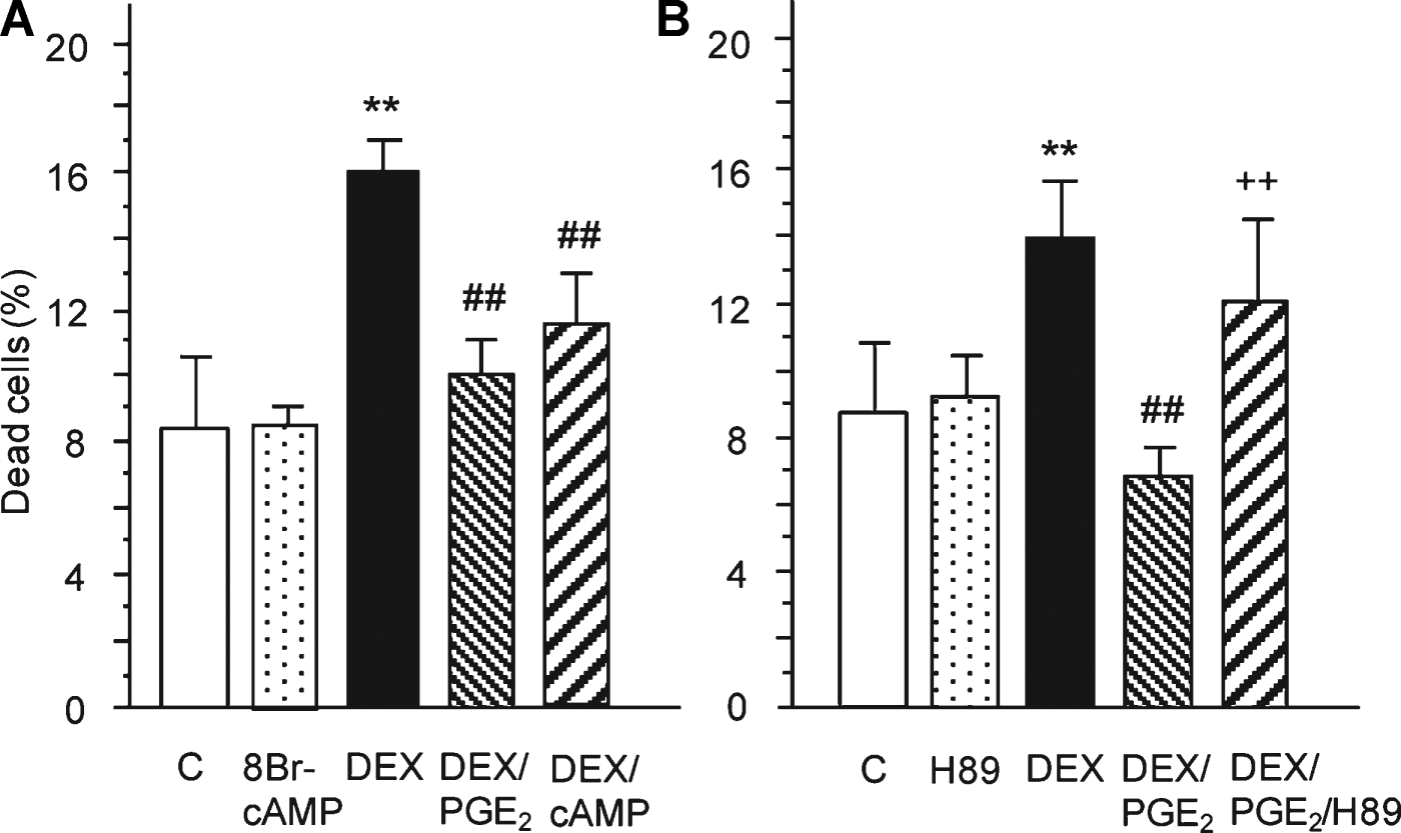
The cAMP/PKA pathway partially mediates the protective effect of PGE_2_ on dexamethasone-induced cell death. To determine the role of this pathway in the protective effects of PGE_2_, MLO-Y4 cells were pretreated with 5 µM of PGE_2_ or 100 µM of 8Br-cAMP for 1 hour, followed by 1 µM of dexamethasone for 6 hours. (*A*) Treatment with 8Br-cAMP significantly inhibited dexamethasone-induced cell death similar to PGE_2_. MLO-Y4 cells were pretreated with H89, a PKA inhibitor, for 30 minutes, followed by treatment with 5 µM of PGE_2_ for 1 hour. The cells then were exposed to 1 µM of dexamethasone for 6 hours. (*B*) The PKA inhibitor H89 partially antagonizes the protective effect of PGE_2_. **p < .01 versus control; ^##^*p* < .01 versus dexamethasone alone; and ^++^*p* < .01 versus dexamethasone with pretreatment of PGE_2_.

EP2 and EP4 receptors are coupled to trimeric G-proteins, and one pathway they can act through is by activation of adenylyl cyclase, which increases intracellular cAMP and results in the activation of protein kinase A (PKA). Therefore, we tested whether an increase in intracellular cAMP would protect MLO-Y4 cells against dexamethasone-induced cell death (Fig. 2). As shown in Fig. 2*A*, 100 µM of 8Br-cAMP was as protective as PGE_2_, and the PKA inhibitor H89 at 5 µM significantly blocked the protective effect of PGE_2_ (Fig. 2*B*).

### The protective effect of PGE_2_ is also mediated via the PI3K/Akt/GSK-3/β-catenin pathway

**Fig. 3:**
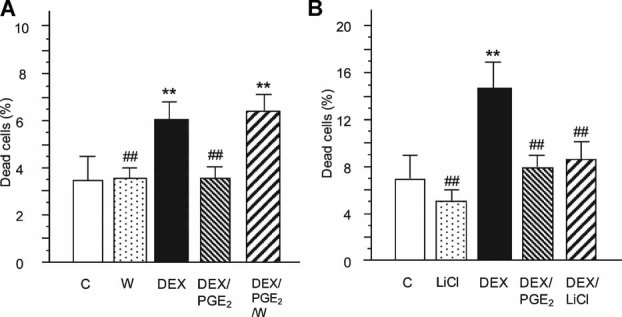
The PI3K/Akt/β-catenin pathway is involved in PGE_2_ protection against dexamethasone-induced apoptosis. (*A*) The protective effects of PGE_2_ against dexamethasone-induced apoptosis are abrogated by wortmanin (designated W), an inhibitor of PI3K. Wortmannin at 10^−6^ M was added. **p < .01 versus control and ^##^*p* < 0.01 versus dexamethasone alone. (*B*) LiCl, known to activate the β-catenin pathway, protects against dexamethasone-induced cell death. MLO-Y4 cells were pretreated with 5 µM of PGE_2_ or 10 mM of LiCl for 1 hour, followed by addition of 1 µM of dexamethasone for 6 hours. Treatment with LiCl inhibited dexamethasone-induced cell death comparable with PGE_2_. **p < .01 versus control and ^##^*p* < .01 versus dexamethasone alone.

PGE_2_ has been shown to activate the PI3K/Akt signaling pathway,(34) stimulate the growth and survival of colon cancer cells through crosstalk with the β-catenin pathway,(35,36) and activate β-catenin signaling in MLO-Y4 osteocyte-like cells.(24,25) In addition, this pathway is activated in response to FFSS in MLO-Y4 osteocyte-like cells.(24,25) Therefore, we sought to determine if PI3K also might be involved in the protective effects of PGE_2_ against dexamethasone-induced apoptosis. The inhibition of PI3K by wortmanin was able to reverse the protective effects of PGE_2_ on dexamethasone-induced apoptosis (Fig. 3*A*). Next, we examined the effect of lithium chloride (LiCl) on MLO-Y4 cell death induced by dexamethasone because LiCl is well known to activate the β-catenin pathway through the inhibition of GSK-3α/β kinase activity. In fact, 10 mM of LiCl showed highly significant protective effects, similar to PGE_2_, against dexamethasone-induced MLO-Y4 cell death (Fig. 3*B*). This suggested that the PI3K and β-catenin pathway mediate the protective effects of PGE_2_.

**Fig. 4:**
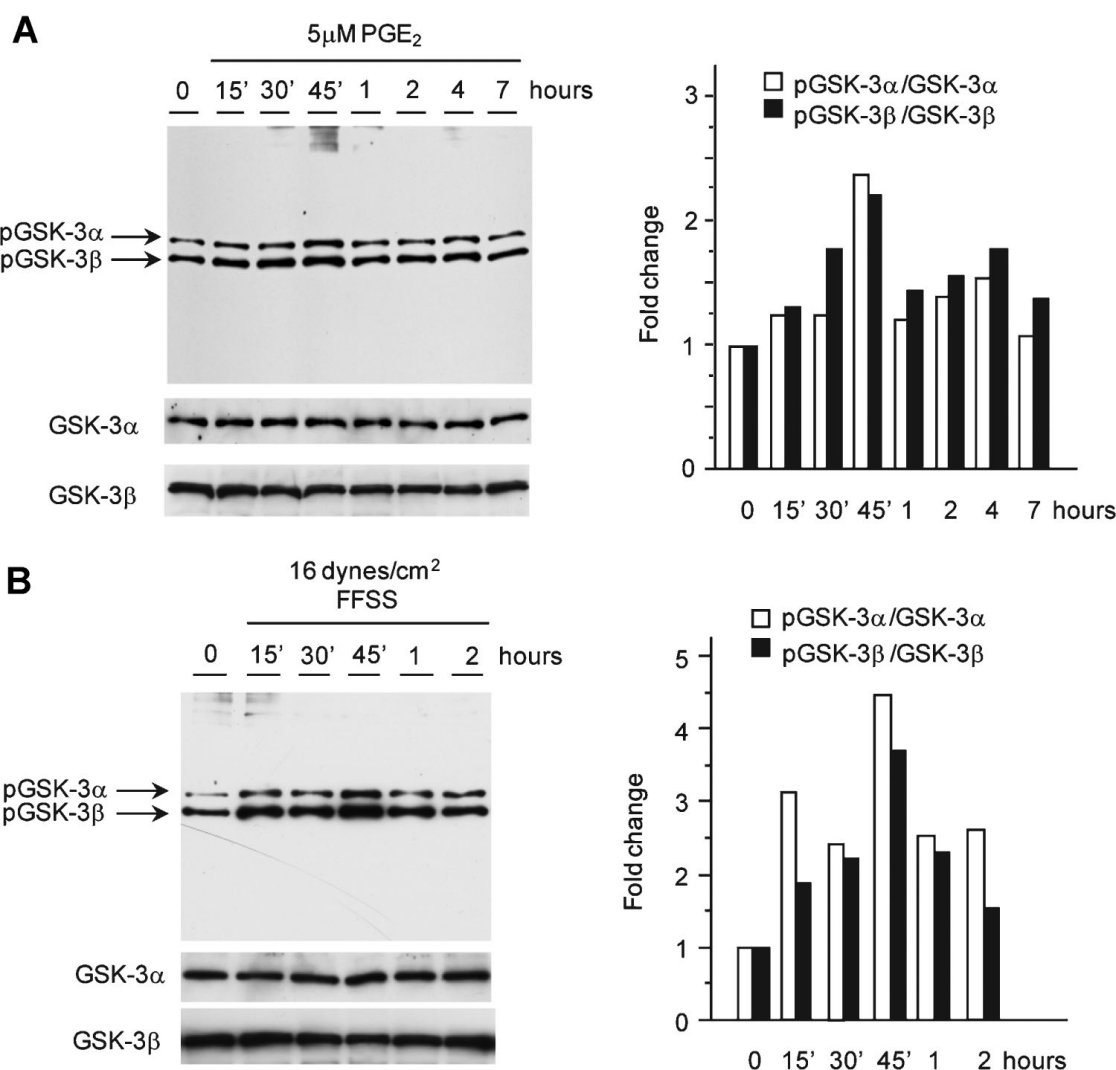
PGE_2_ and FFSS similarly increase the phosphorylation of GSK-3α and -β, a mediator of β-catenin activation. Western blot analysis and quantitation of phospho-GSK-3α and -β was performed in MLO-Y4 cells with exogenous addition of PGE_2_ and with exposure to FFSS. (*A*) MLO-Y4 cells treated with 5 µM of PGE_2_ also showed a maximal induction of phosphorylation of α and β at 45 minutes. After 2 hours of 16 dyn/cm^2^ FFSS, the cell lysates were harvested at 15, 30 45, 60, and 120 minutes. (*B*) FFSS also induced phosphorylation of both α and β, with the highest induction being at 45 minutes after cessation of shear stress. Semiquantitative analysis of band intensity was performed using the intensity of total GSK-3α and -β bands for normalization of the phosphorylated bands. Both FFSS and PGE_2_ increased the phosphorylation of both of GSK-3α and -β maximally at 45 minutes after application of stimulus.

Next, we tested whether both FFSS and PGE_2_ could lead to increased phosphorylation/inactivation of GSK-3α/β (Fig. 4). In fact, 5 µM of PGE_2_ effectively increased the phosphorylation of GSK-3α and GSK-3β by approximately twofold at about 45 minutes (Fig. 4*A*). FFSS at 16 dyn/cm^2^ increased GSK-3α/β phosphorylation similar to PGE_2_. Western blot analysis revealed that FFSS was able to increase the phosphorylation of both GSK-3α and GSK-3β isoforms by approximately three- to fourfold at 45 minutes compared with the negative control (Fig. 4*B*). Both FFSS and PGE_2_ showed similar kinetics, reaching a peak effect at 45 minutes, the same time point showing maximal phosphorylation induced by FFSS.

**Fig. 5:**
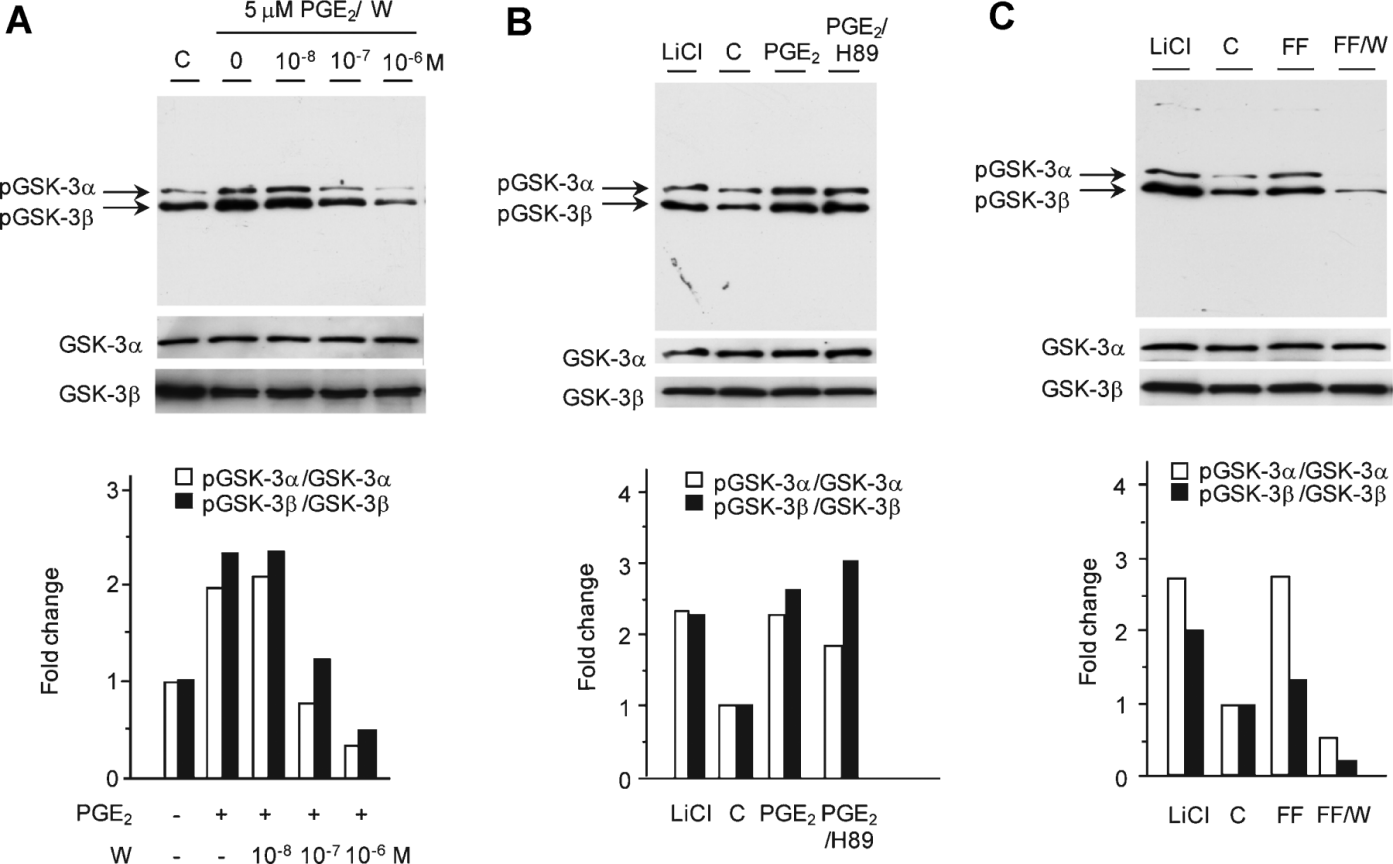
The PI3K inhibitor wortmannin prevents the phosphorylation of GSK-3α and -β by PGE_2_ and FFSS, whereas the PKA inhibitor H89 had no effect. (*A*) Western blot analysis of phospho-GSK-3 in MLO-Y4 cells pretreated with varying concentrations of wortmannin for 30 minutes before application of 5 µM of PGE_2_ for 45 minutes. Wortmannin at 10^−6^ and 10^−7^ M dramatically inhibited GSK-3α and -β phosphorylation, as induced by PGE_2_. (*B*) Wortmannin prevents the phosphorylation of GSK-3 by FFSS, similar to PGE_2_. MLO-Y4 cells were pretreated with 1 µM of wortmannin for 1 hour before being subjected to FFSS of 16 dyn/cm^2^ for 45 minutes. LiCl was used as a positive control. (*C*) H89 had no effect on PGE_2_-induced phosphorylation of GSK-3α and -β.

To validate that PI3K is upstream of GSK-3β inhibition, cells exposed to either PGE_2_ or to FFSS were treated with the PI3K inhibitor wortmannin. The inhibition of PI3K reversed the protective effects of PGE_2_ on dexamethasone-induced apoptosis (Fig. 3*A*). Wortmannin at 10^−6^ to 10^−7^ M inhibited the phosphorylation of GSK-3 induced by 5 µM of PGE_2_ dose-dependently, with maximum inhibition at 10^−6^ M (Fig. 5*A*). Similar to PGE_2_, the phosphorylation of GSK-3α/β induced by FFSS also was blocked by addition of 1 µM of wortmannin (Fig. 5*B*). We have shown previously that PGE_2_ increases intracellular cAMP and activates PKA in MLO-Y4 cells.(32) PKA kinase has been shown to mediate phosphorylation of GSK-3α/β.(34,37,38) The PKA inhibitor H89 at 5 µM completely blocked PKA activity in MLO-Y4 cells(39) but did not inhibit the phosphorylation of GSK-3α/β (Fig. 5*C*). This suggests that the effects of PGE_2_ on GSK phosphorylation are mediated through PI3K but not PKA activity.

### FFSS, LiCl, and PGE_2_ promote nuclear translocation, whereas dexamethasone inhibits stabilization of β-catenin

**Fig. 6:**
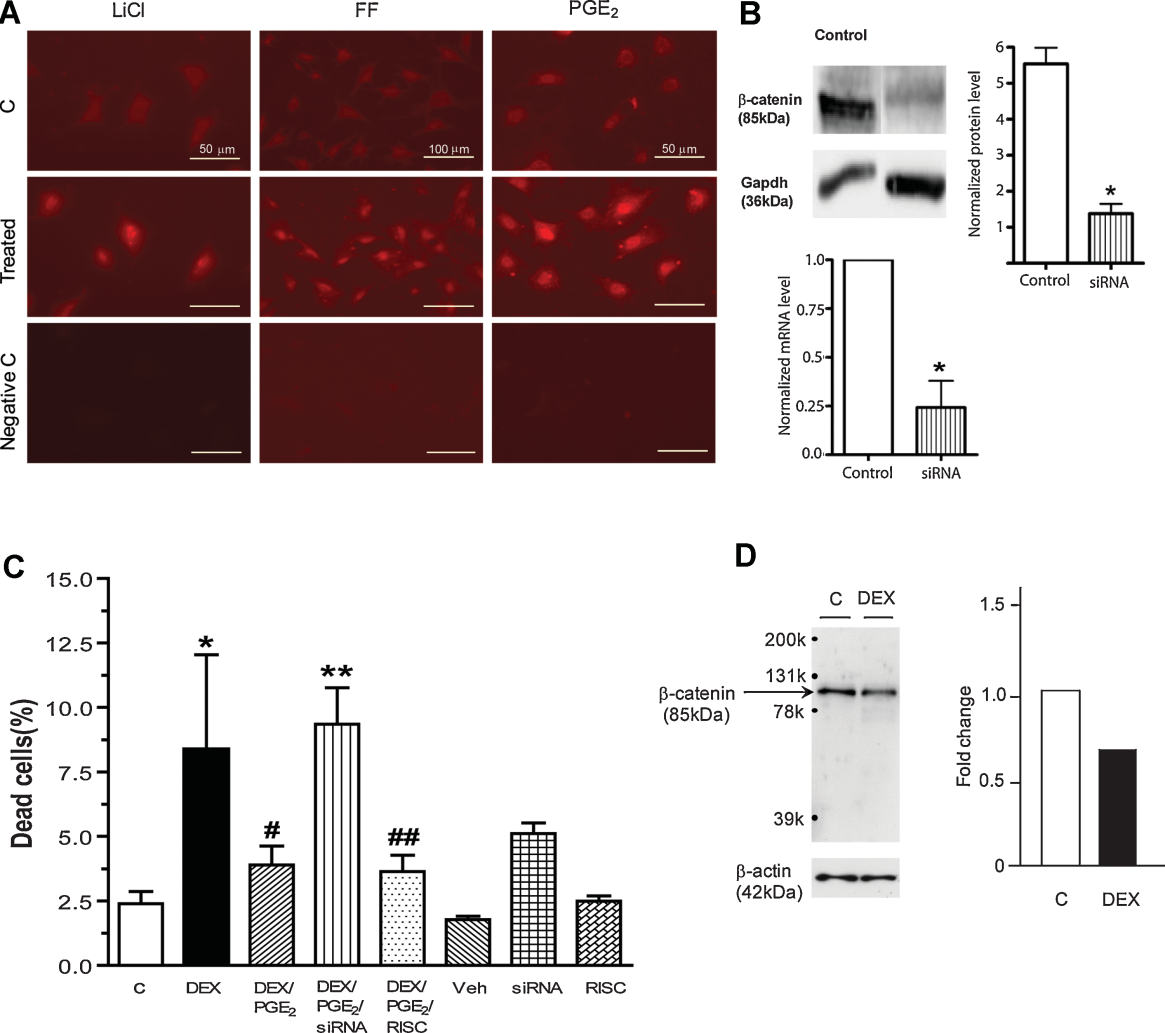
LiCl, FFSS, and PGE_2_ stabilize and activate the nuclear translocation of β-catenin, whereas dexamethasone reduces β-catenin protein expression. Cells were treated with 10 mM of LiCl, 16 dyn/cm^2^ of FFSS, or 5 µM of PGE_2_ for 2 hours, followed by fixation, and then were subjected to immunofluorescent immunostaining using antibody against β-catenin, as described under “Materials and Methods.” (*A*) FFSS and PGE_2_ treatment induces β-catenin stabilization and nuclear translocation comparable with that of LiCl. (*B*) Transfection of MLO-Y4 cells with *β-catenin* siRNA reduced both mRNA and protein levels by 75% to 80%. (*C*) β-Catenin silencer siRNA reversed the protective effect of PGE_2_, whereas RISC-free negative control failed to block the protective effect. Cells were transiently transfected with siRNA oligonucleotides targeting β-catenin and RISC-free negative control for 48 hours, followed by 5 µM of PGE_2_ for 2 hours. The cells then were exposed to 1 µM of dexamethasone for 6 hours. (*D*) Western blot analysis of β-catenin in MLO-Y4 cells treated with 10^−6^ M of dexamethasone was performed. A reduction in β-catenin protein was observed. *p < .05 versus control (designated C). ^#^*p* < .05 versus dexamethasone alone. **p < .05 versus control or versus dexamethasone/PGE_2_. ^##^*p* < .05 versus dexamethasone/PGE_2_/siRNA or versus dexamethasone alone.

Both FFSS and PGE_2_ induced β-catenin nuclear translocation, similar to LiCl (positive control), as shown in Fig. 6. Clearly, nuclear translocation of β-catenin occurs in MLO-Y4 cells subjected to either FFSS or to PGE_2_, as indicated by the bright nuclear staining (Fig. 6*A*). This shows that both PGE_2_ and FFSS target β*-*catenin to the nucleus. Next, siRNA knockdown experiments were performed using siRNA to β-catenin. Western blotting showed that β-catenin protein was significantly reduced fourfold at 48 hours after transfection (Fig. 6*B*). qPCR showed that *β-catenin* mRNA decreased 86% compared with the RISC-free negative control at 24 hours after transfection (Fig. 6*B*). Cells were transiently transfected with siRNA oligonucleotides targeting β-catenin and RISC-free negative control for 48 hours, followed by incubation with 5 µM of PGE_2_ for 2 hours (Fig. 6*C*). The cells then were exposed to 1 µM of dexamethasone for 6 hours. β-catenin silencer siRNA reversed the protective effect of PGE_2_, whereas the RISC-free negative control had no significant effect. Transfection of siRNA oligonucleotides alone had no significant effect. Vehicle, siRNA alone, or RISC alone had no significant effect. Knockdown of β-catenin blocked the protective effect of PGE_2_ against the dexamethasone-induced apoptosis.

Next, Western blot analysis of β-catenin in MLO-Y4 cells treated with 10 mM LiCl, 5 µM PGE_2_, and FFSS at 16 dyn/cm^2^ was performed to determine if these treatments altered protein levels. No major change in total protein expression was observed compared with control (data not shown). However, dexamethasone decreased the amount of β-catenin protein approximately 40% compared with control (Fig. 6*D*).

To determine if dexamethasone has an effect on upstream mediators of the β-catenin pathway, we investigated the effects of dexamethasone on expression of PGE_2_ synthase (COX-2) and the EP receptors (Table 3). The relative expression of mRNA for the cyclooxygenase isoenzymes COX-1 and -2 and the PGE_2_ receptors EP1 to EP4 in MLO-Y4 cells treated with 1 µM of dexamethasone for 4 and 24 hours using cDNA microarray analysis is shown in Table 3A. *COX-2* mRNA was inhibited by dexamethasone treatment, 2- and 15-fold lower at 4 and 24 hours, respectively, compared with control. Dexamthasone decreased EP2 receptor mRNA levels to a greater extent than the other three receptors, approximately threefold lower than control at 24 hours. The positive controls in this experiment were GILZ (glucocorticoid-induced leucine zipper) and FKBP51 (FK-506-binding protein), which have been reported previously to be increased by glucocorticoids.(40,41) Validation of the gene array approach by qRT-PCR showed mRNA expression of COX-2 and EP2 receptor in MLO-Y4 cells to be significantly reduced by dexamethasone at each time point (6, 24, and 48 hours) (Table 3B).

**Table 3A:** Dexamethasone Inhibits the Expression of COX-2 and the EP2 Receptor Genes and Downstream Targets of the β-Catenin Pathway

Gene	4 hours	24 hours
*Ptgs1* (COX-1)	0.82	0.66
*Ptgs2* (COX-2)	0.43	0.06^a^
*Ptger1* (EP1 receptor)	0.80	1.08
*Ptger2* (EP2 receptor)	0.80	0.34^a^
*Ptger3* (EP3 receptor)	0.89	0.84
*Ptger4* (EP4 receptor)	0.74	0.92
*Wisp1*	1.03	0.52
*Vegf*	0.73	0.42
*Connexin 43*	1.01	0.53
*Gilz*	6.83	4.60
*Fkbp51*	6.13	6.19

*Note:* MLO-Y4 cells were treated with 1 µM dexamethasone for 4 or 24 hours, and mRNA levels measured using mouse genome 430A 2.0 array chips according to the manufacturer's protocols. Expression is shown as fold induction based on the expression level in control, nontreated MLO-Y4 cells at 4 and 24 hours, respectively.

a Significant changes confirmed by real-time quantitative PCR.

**Table 3B:** Real-Time PCR Data Showing That Both COX-2 and EP2 Are Reduced with Dexamethasone Treatment Over 6, 24, and 48 Hours

	COX-2 relative to control (Ctrl)	EP2 receptor relative to control (Ctrl)
Ctrl—6 h	1.0 (0.88–1.14)	1.0 (0.64–1.57)
Dex—6 h	0.26 (0.19–0.35)	0.11 (0.08–0.16)
Ctrl—24 h	1.0 (0.85–1.18)	1.0 (0.63–1.58)
Dex—24 h	0.19 (0.14–0.24)	0.23 (0.17–0.31)
Ctrl—48 h	1.0 (0.84–1.18)	1.0 (0.79–1.27)
Dex—48 h	0.04 (0.03–0.05)	0.22 (0.18–0.26)

## Discussion

**Fig. 7:**
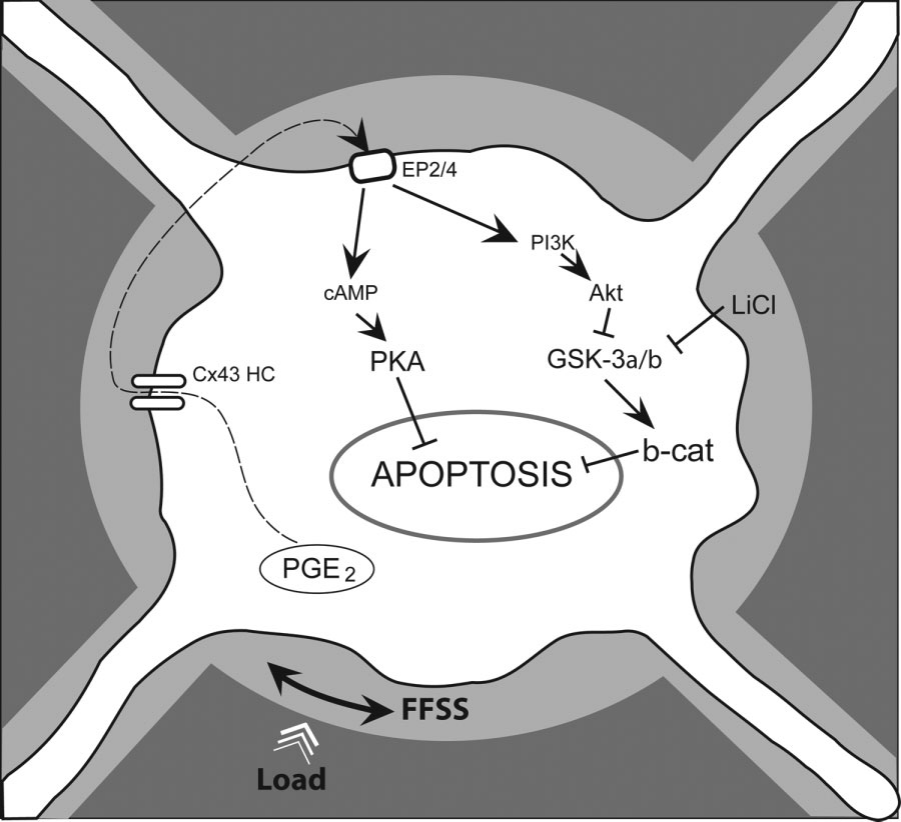
A diagram showing the means whereby mechanical loading (ɛ) prevents apoptosis. Mechanical loading, in the form of FFSS, induces the release of prostaglandin (PGE_2_) through connexin 43 hemichannels (Cx43 HCs), as shown previously by Cherian and colleagues(32) to have both autocrine and paracrine effects through EP2 and EP4 receptors. These receptors not only signal through the traditional cAMP/PKA pathway to reduce apoptosis but also signal through the PI3k/Akt/GSK-3/β-catenin pathway. Lithium chloride (LiCl) also blocks apoptosis. The same mechanism and pathways were shown to be responsible for the transcription of Cx43 and increased gap junction function.(25) For additional information on the potential for crosstalk between these two pathways and role in bone cell function, see the review by Bonewald and Johnson.(60)

We describe for the first time a mechanism that connects fluid-flow shear stress (FFSS)–induced release of PGE_2_ with downstream signaling pathways that protect osteocytes against dexamethasone-induced apoptosis. Shear stress was shown to prevent apoptosis induced by dexamethasone in MLO-Y4 osteocyte-like cells by inducing PGE_2_ release and its subsequent binding to EP2 and EP4 receptors activating both the cAMP/PKA and PI3K/Akt/GSK-3α/β/β-catenin pathways. Dexamethasone inhibited β-catenin stabilization and the expression of downstream target genes in addition to inhibiting genes responsible for prostaglandin production and signaling, *COX-2* and the *EP2 receptor*. An important function for prostaglandin has been identified, which is as an antiapoptotic agent for osteocytes that provides protection against glucocorticoid-induced apoptosis. The results of our studies are illustrated in the model shown in Fig. 7.

The osteocyte lacunocanalicular system is filled with bone fluid, and it is hypothesized that it is the movement of this fluid that maintains osteocyte viability by preventing hypoxia in these cells deeply embedded in a mineralized matrix.(42,43) In addition to maintaining osteocyte viability, movement of the bone fluid most likely induces shear stress on the cell membrane of the cell body and along the dendritic process traveling through canaliculi.(44) This shear stress is thought to be the means whereby the cells sense load on the skeleton.(45) Mechanical loading is well known to maintain osteocyte viability, and physiologic levels of mechanical loading were shown to prevent apoptosis of osteocytes in vivo.(17) Alternatively, reduced mechanical loading increases the number of apoptotic osteocytes.(16) In vitro, mechanical loading reduces the number of osteocytes undergoing apoptosis induced by serum starvation(46) and by dexamethasone,(18) but the molecular mechanisms have not been reported. These studies suggest that one potential agent produced by osteocytes in response to mechanical loading is PGE_2_, and our data suggest that it functions as an osteocyte viability factor.

Evidence is emerging that PGE_2_ can function as either a pro- or an antiapoptotic factor. It has been reported that PGE_2_ can function as a protective factor in several types of cells, such as epithelial cells, dendritic cells, and neurons.(47–49) In bone cells, PGE_2_ was shown to exert an antiapoptotic effect on bone marrow stromal cells and periosteal cells, thereby increasing their number and subsequent osteoblastic differentiation.(49,50) There are also opposing reports showing that PGE_2_ can induce apoptosis in several types of cells, such as articular chondrocytes.(51) Thus the effect of PGE_2_ in cell survival or apoptosis may depend on cell and/or tissue type.

PGE_2_ can bind to four subtypes of cell surface receptors, designated EP1 through EP4.(33) EP2 and EP4 receptors activate and signal through adenylyl cyclase, EP1 activates phospholipase C, and EP3 actually inhibits adenylyl cyclase.(33,52,53) By using agonists and antagonists to each receptor, we observed that the effects of PGE_2_ were mediated mainly through the EP2 receptor, with less activation of the EP4 receptor. These same receptors have been shown to mediate the effects of PGE_2_ on survival of bone marrow stromal cells and periosteal cells.(49,50) Activated adenylate cyclase via EP2 and EP4 receptors increases cAMP, leading to activation of PKA.(53) In this study, the use of a cAMP analogue, 8Br-cAMP, and a PKA inhibitor, H89, showed that the cAMP/PKA pathway is involved in the protective effects of PGE_2_. This suggests that activation of the cAMP/PKA pathway also plays a critical role in maintaining osteocyte viability.

However, in this study, the protective effects of PGE_2_ were not completely mediated through the activation of the cAMP/PKA signaling cascade. PGE_2_ also can activate multiple signaling cascades, including the MAPK, NF-κB, and PI3K/Akt pathways,(54–56) and recently, it has been reported to crosstalk with and activate the β-catenin signaling pathway, well known to play an important role in cell survival.(35) In colon cancer, PGE_2_ activates the EP2 receptor, leading to activation of PI3K and the protein kinase Akt by free G-protein βγ subunits and the direct association of the G-protein α_s_ subunit with axin. This leads to collapse of the degradation complex and β-catenin nuclear accumulation responsible for colon cancer cell viability.(35) The protective effects of PGE_2_ in neurons also have been reported to occur by transactivation of β-catenin.(57)

In this study, we observed that activation of the β-catenin pathway by LiCl resulted in the rescue of osteocytes from cell death. The use of the PI3K inhibitor wortmannin also prevented the phosphorylation of GSK-3, partially abrogating the antiapoptotic effects of PGE_2_. Together these data indicate that the PI3K/Akt/GSK-3/β-catenin pathways also play a key role in PGE_2_-mediated osteocyte survival. Similar observations were made with regard to the antiapoptotic actions of PGE_2_ in neurons.(57) Therefore, the protective effects of PGE_2_ appear to be mediated through multiple signaling cascades.

Previous studies in transgenic mice carrying the G171V or HBM (high-bone-mass) mutation in *Lrp5* have shown decreased osteoblast and osteocyte apoptosis in the bones of these mice.(58) The *Lrp5* coreceptor that binds Wnt and regulates the Wnt/β-catenin signaling pathway is absolutely required for new bone formation in response to mechanical loading.(59) The data presented here extend these in vivo observations by demonstrating that a normal function of β-catenin signaling in osteocytes is maintenance of cell viability. Our data further illustrate the complex and multiple interactions (Fig. 7) that exist within bone cells between various signaling pathways and that understanding osteocyte function will require a thorough knowledge of how these pathways are coordinately regulated in response to various perturbations.

In summary, PGE_2_ produced by osteocytes in response to mechanical strain shows a protective function against glucocorticoid through activation of several signaling cascades. Since osteocyte cell death is a potential mediator of targeted osteoclast recruitment, preservation of osteocyte viability is another area of investigation for the prevention or attenuation of primary and secondary osteoporosis and other bone-related diseases. It will be important to determine if increased mechanical loading alone or in combination with therapeutics will reduce or prevent the detrimental effects of glucocorticoid on osteocyte viability and bone integrity.
